# Presence of variable extrathoracic airflow limitation in patients with a negative methacholine challenge test

**DOI:** 10.1186/s13223-023-00860-w

**Published:** 2023-11-29

**Authors:** Zane Z. Elfessi, Sarah Zavala, Israel Rubinstein

**Affiliations:** 1 Emergency Medicine, Jesse Brown VA Medical Center, Chicago, IL 60612 USA; 2 Department of Medicine, Jesse Brown VA Medical Center, Chicago, IL 60612 USA; 3 Research Services, Jesse Brown VA Medical Center, Chicago, IL 60612 USA; 4 University of Illinois College of Pharmacy in Chicago, Chicago, IL 60612 USA; 5 Division of Pulmonary, Critical Care, Sleep, and Allergy Medicine (M/C 719), Department of Medicine, University of Illinois College of Medicine in Chicago, 840 South Wood Street, Chicago, IL 60612 USA

**Keywords:** Spirometry, FEF_50_/FIF_50_, Obstructive sleep apnea, Inducible laryngeal obstruction, Cough, Obesity

## Abstract

**Purpose:**

Determine whether variable extrathoracic airflow limitation (VEAL) is observed in patients with negative methacholine challenge tests (MCT).

**Methods:**

Electronic medical records of patients undergoing MCT at Jesse Brown VA Medical Center between January 2017 and December 2019 were reviewed. Only patients with negative MCT were selected. Pertinent demographic, clinical, and pulmonary function tests (PFT) and MCT data were abstracted from each record. Spirometric flow-volume loops recorded during each test were inspected by one co-author to determine the first inhaled methacholine concentration at which FEF_50_/FIF_50_ was either > 1 or further increased if baseline FEF_50_/FIF_50_ after nebulized saline (vehicle) already exceeded 1. Student’s t-test was used for statistical analysis. P < 0.05 was considered statistically significant.

**Results:**

One hundred and twenty-seven consecutive patients with normal baseline PFT and negative MCT were identified. Thirteen patients (10.2%) had negative MCT and FEF_50_/FIF_50_ > 1 after testing. They were predominately obese (BMI, 31.3 ± 6.6), non-smoking (10), White (8) males (9) aged 51.3 ± 14.1 years (mean ± SD) referred for symptoms suggestive of asthma (n = 7) or for chronic cough (n = 6). Five had obstructive sleep apnea, three gastroesophageal reflux disease, and two chronic rhinosinusitis. FEF_50_/FIF_50_ increased significantly from 0.72 ± 0.21 after nebulized saline (vehicle) to 1.21 ± 0.13 after inhaled methacholine (p < 0.001). Median inhaled methacholine concentration eliciting these responses was 1.0 mg/mL (range, 0.25–16 mg/mL).

**Conclusions:**

VEAL is observed in a subset of patients with a negative MCT. This phenomenon should be recognized and reported to the referring healthcare providers and its clinical significance addressed as indicated.

## Introduction

Methacholine challenge testing (MCT) is a form of bronchoprovocation testing, which uses the acetylcholine derivative methacholine to induce bronchoconstriction. In this test, methacholine is administered via nebulization in increasing concentrations ranging from 0.016 to 16 mg/mL, in two to four-fold dilutions. Forced expiratory volume in 1 s (FEV_1_) is measured after each successive dose and the test is stopped and considered positive when the FEV_1_ drops by more than 20% from baseline—considered the provocation dose (PC_20_). A negative MCT, is defined by a no response to the highest concentration of methacholine administered [1]. Current guidelines on performance of methacholine challenge test (MCT) in adults are limited to the expiratory portion of the flow-volume curve recorded during spirometry [1, 2]. However, previous studies have shown that inhaled methacholine could concomitantly affect the inspiratory portion of the flow-volume curve suggesting the presence of variable extrathoracic airflow limitation (VEAL) [3–5]. Whether this response is also observed and reported in patients with negative MCT is uncertain.

Conceivably, isolated inspiratory flow limitation as assessed by maximum expiratory to inspiratory flows at 50% of forced vital capacity ratio (FEF_50_/FIF_50_) observed during a negative MCT could guide healthcare providers to consider alternative upper airway disorders associated with laryngeal hyperresponsiveness. These conditions, such as obstructive sleep apnea, reflux disease, inducible laryngeal obstruction and chronic rhinosinusitis, could then be treated accordingly [6–8].

Therefore, the purpose of this study was to begin to address this issue by determining whether VEAL is observed and reported in patients with negative MCT at our facility.

## Methods

The electronic health records (EHR) of patients undergoing MCT at Jesse Brown VA Medical Center (JBVAMC), Chicago, Illinois, between January 2017 and December 2019 were reviewed. Only patients with negative MCT according to the American Thoracic Society guidelines were selected [1].

Pertinent demographic, clinical, pulmonary function tests (PFT) and MCT data were abstracted from each record. All PFT and MCT data were reviewed by one co-author (ZZE). Spirometric flow-volume loops recorded during each test were inspected to determine the first inhaled methacholine concentration at which FEF_50_/FIF_50_ was either > 1 or further increased if baseline FEF_50_/FIF_50_ after nebulized saline (vehicle) already exceeded 1.

### Data and statistical analyses

Data are reported as means and standard deviation where appropriate. Student’s t-test was used for statistical analysis. P < 0.05 was considered statistically significant.

## Results

**Fig. 1:**
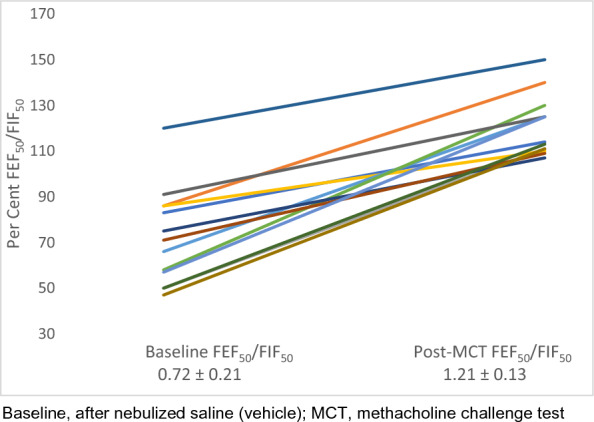
Baseline* and post-methacholine challenge FEF_50_/FIF_50_ (n = 13). Baseline, after nebulized saline (vehicle); *MCT* methacholine challenge test

A total of 139 MCTs were performed during the 3-year study period of which 127 were negative. Thirteen patients (10.2%) with negative MCTs had FEF_50_/FIF_50_ > 100% post-MCT (Table 1). In twelve patients, FEF_50_/FIF_50_ exceeded 1 after testing while in one patient with baseline FEF_50_/FIF_50_ > 1 it further increased after testing (Fig. 1). These findings were not noted in the report sent to the referring healthcare providers.

**Table 1:** Patient characteristics

	Patients (n = 13)
Age, years	51.3 ± 14.1
Males, n (%)	9 (69.2)
Race, n (%)	
African American	5 (38.5)
White	8 (61.5)
BMI, kg/m^2^	31.3 ± 6.6
Reported smoking history, n (%)	
Current	2 (15.4)
Past	1 (7.7)
Never	10 (76.9)

Data are means ± standard deviation

*BMI* body mass index, *OSA* obstructive sleep apnea

Patients were predominately obese (BMI, 31.3 ± 6.6), non-smoking (n = 10), White (n = 8) males (n = 9) aged 51.3 ± 14.1 years who were referred for evaluation of symptoms suggestive of asthma (n = 7) or for chronic cough (n = 6). Five had physician-diagnosed obstructive sleep apnea (OSA), three gastroesophageal reflux disease, and two chronic rhinosinusitis (Tables 1 and 2). In three patients with a negative MCT, a presumptive diagnosis that may underlie VEAL was not established.

**Table 2:** Methacholine challenge test data

	Patient cohort (N = 13)
Baseline PFT	
FVC, L	3.97 ± 0.98
FVC % predicted	98 ± 12.32
FEV_1_, L	3.03 ± 0.80
FEV_1_% predicted	92.55 ± 12.01
FEV_1_/FVC	0.76 ± 0.08
D_LCO_, mL/min/mmHg	23.67 ± 6.14
D_LCO_, % predicted	83.02 ± 17.89
FEF_50_/FIF_50_, %	72 ± 21
^a^PFT after methacholine challenge test	
FVC, L	3.89 ± 0.92
FVC % predicted	96.6 ± 12.32
FEV_1_, L	3.16 ± 0.83
FEV_1_% predicted	99.7 ± 14.31
FEV_1_/FVC	0.81 ± 0.06
FEF_50_/FIF_50_	1.21 ± 0.13
Increase in FEF_50_/FIF_50_ from baseline	0.4 ± 0.14
Fall in FIF_50_ > 20% from baseline, n (%)	11 (84)
Methacholine concentration^a^, mg/mL, median (range)	1.0 (0.25–16)

Data are means ± standard deviation

PFT: pulmonary function tests; FVC: forced vital capacity; FEV_1_: forced expiratory volume in one second; FEF_50_: forced expiratory flow rate at 50% vital capacity; FIF_50_: forced inspiratory flow rate at 50% vital capacity; D_LCO_: diffusion capacity of the lungs for carbon monoxide

^a^The first methacholine concentration at which FEF_50_/FIF_50_ was either > 100% or further increased if baseline FEF_50_/FIF_50_ after nebulized saline (vehicle) already exceeded 100%

Mean FEF_50_/FIF_50_ increased significantly from 0.72 ± 0.21 after nebulized saline (vehicle) to 1.21 ± 0.13 after inhaled methacholine (Fig. 1). The median inhaled methacholine concentration eliciting this response was 1.0 mg/mL (range, 0.25–16 mg/mL) (Table 2). Using Kelso et al. definition [3], we found that 11 out of 13 patients had a clinically significant decrease in the FIF_50_ > 20% after MCT.

## Discussion

The new finding of this study is that VEAL observed in a small proportion of patients with negative MCT at our facility was not interpreted nor reported to the referring healthcare providers. Conceivably, these spirometric data could indicate the presence of alternative disorders associated with laryngeal hyperresponsiveness that should then be pursued and treated accordingly [6–11]. Hence, we propose that FEF_50_/FIF_50_ recorded during MCT should be interpreted and reported to the referring healthcare providers and its clinical implications addressed as indicated.

Kelso et al. [3] showed that in fourteen of seventy-six consecutive patients with negative MCT (18%) FIF_50_ decreased by 20 to 35% from baseline suggesting the presence of VEAL. However, baseline and post-MCT FEF_50_/FIF_50_ data were not reported. Moreover, criteria for a positive inspiratory challenge during MCT of ≥ 20% fall in FIF_50_ from baseline chosen by these authors have not been published so far [1, 2].

To the best of our knowledge, VEAL reported in our patients with OSA and a negative MCT has not been previously described in the literature. To that end, Lin et al. [12] found positive MCT in four of sixteen patients with OSA but did not report FEF_50_/FIF_50_ data in those with negative MCT. Whether patients with OSA and VEAL observed during negative MCTs represent a distinct phenotype of upper airway dysfunction in this disorder remains to be determined. To that end, obesity, a distinct feature in patients with OSA, is associated with tidal flow limitation due to reduced functional residual capacity and expiratory reserve volume [13]. Conceivably, this phenomenon could result in higher nebulized methacholine dose delivered to the upper airway of obese patients leading to local, non-selective muscarinic receptor activation and VEAL. Further studies are warranted to support or refute this hypothesis.

Several limitations of this study are notable. It was a small, retrospective, single site study comprised predominantly of white obese males. Hence, generalizability of our observations is limited. Therefore, we propose that a larger, prospective, multi-site study should be conducted to determine the prevalence of VEAL in patients with negative MCT and to unravel upper airway disorders associated with this phenomenon.

In summary, we found that VEAL is observed in some patients with a negative MCT. This phenomenon should be interpreted and reported to the referring healthcare providers and its clinical implications addressed as indicated.

## Data Availability

The datasets during and/or analyzed during the current study and available from the corresponding author on reasonable request.
